# Supplementary material for: CRISPR-Cas9-Mediated Cytosine Base Editing Screen for the Functional Assessment of CALR Intron Variants in Japanese Encephalitis Virus Replication
Source: Int J Mol Sci. 2023 Aug 28;24(17):13331. doi: 10.3390/ijms241713331 (PMC10487596; doi:10.3390/ijms241713331)
Supplement: Supplementary file 1 [file ijms-24-13331-s001.zip › ijms-2500390-supplementary.pdf]

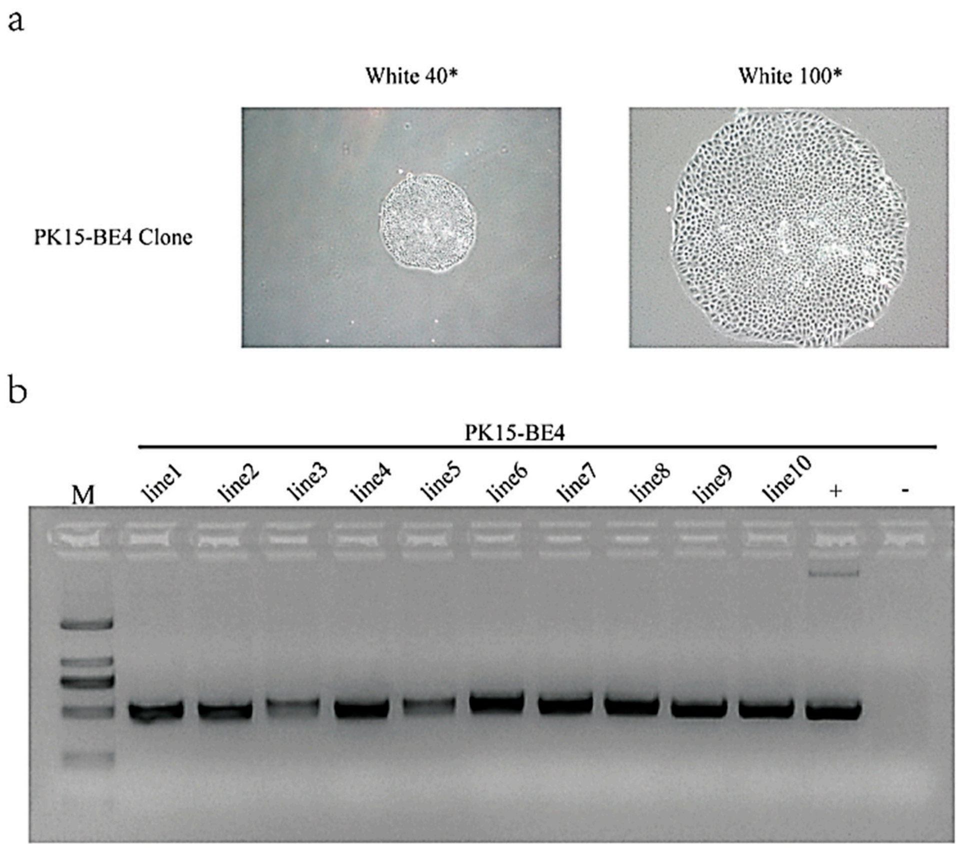

Figure S1. The generation and identification of PK15-BE4 clone lines. (a) The Morphology of PK15-BE4 cell clone before being picked up. (b) PCR amplification of the partial sequence of Cas9. M, marker; +, positive control (plasmid); -, negative control (wild-type PK15 DNA). Each lane refers to a cell line, respectively.

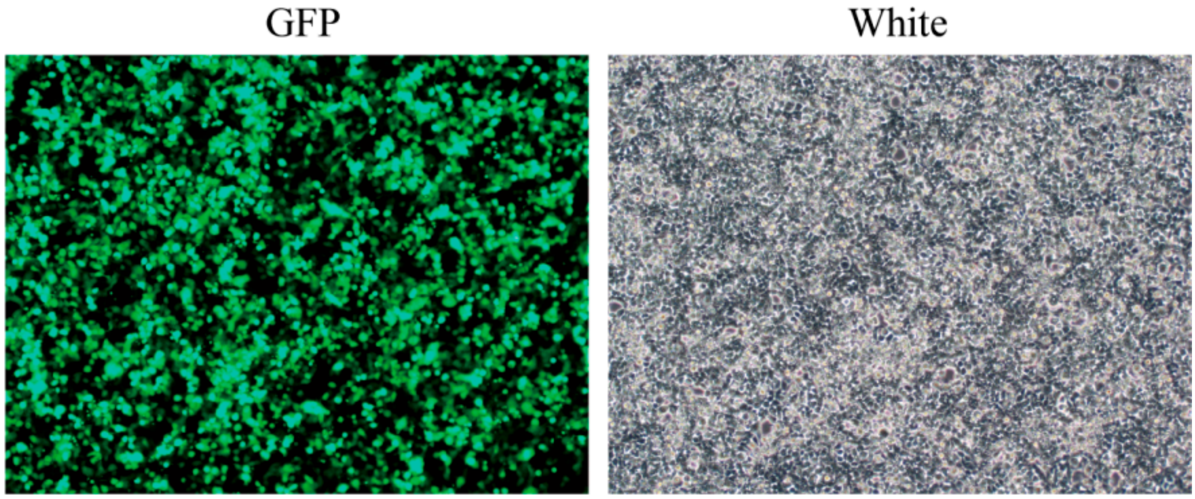

Figure S2. The efficiency of packaging of the sgRNA plasmid library.

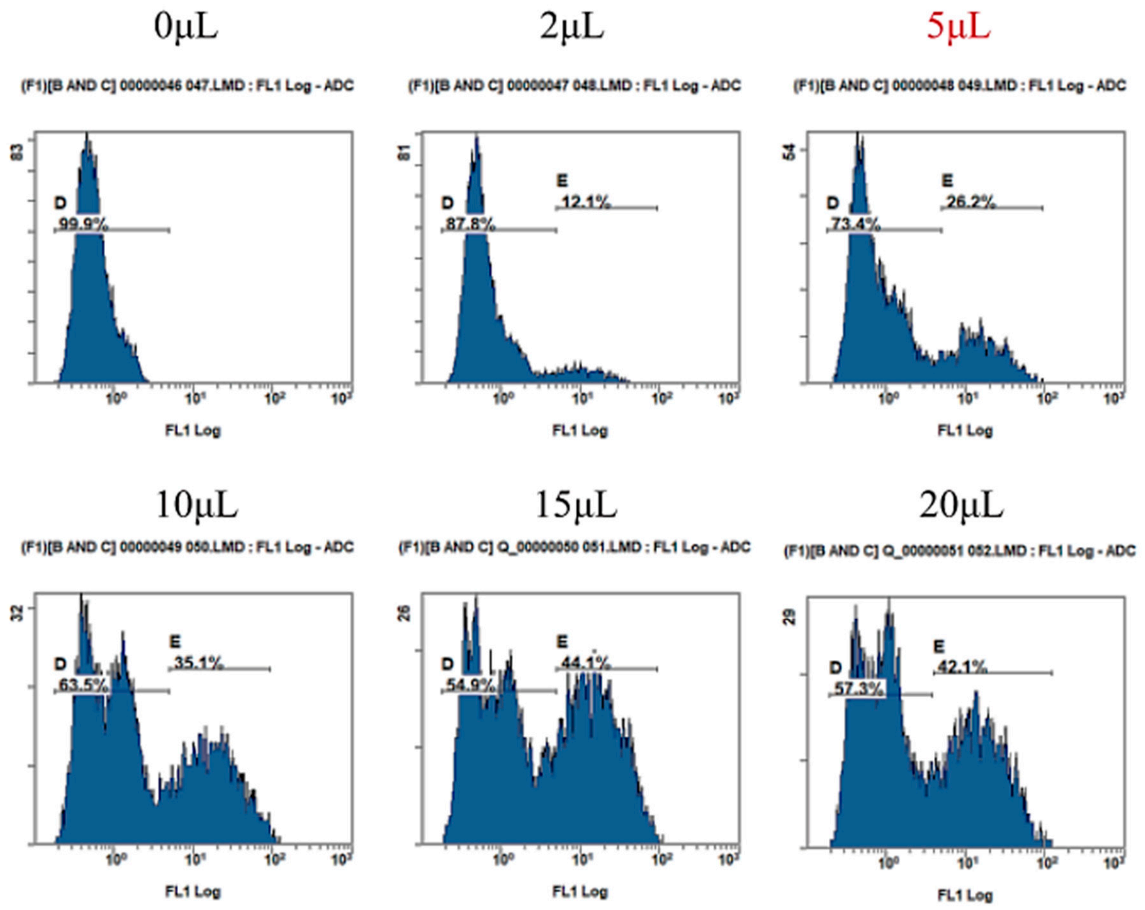

Figure S3. The optimal dose of the library lentivirus for the PK15-BE4 cells by FACS. The PK15-BE4 cells were infected with 6 different doses (μL) (0, 2, 5, 10, 15, 20), and the optimal dose with MOI < 0.3 was determined by FACS.

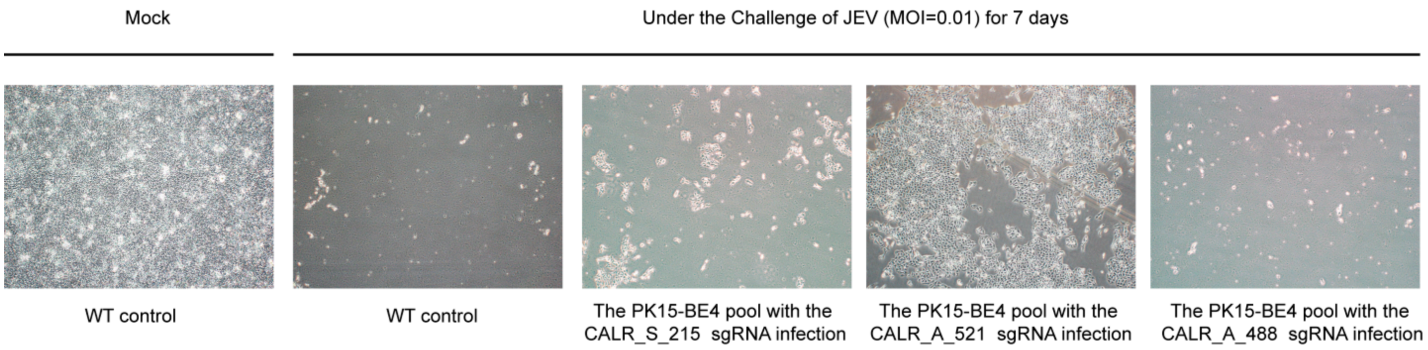

Figure S4. Comparison of the ability of three PK15-BE4 cell pools with the individual (top3) sgRNA infection and wild-type cells to resist JEV-induced cell death using cell proliferation assays.

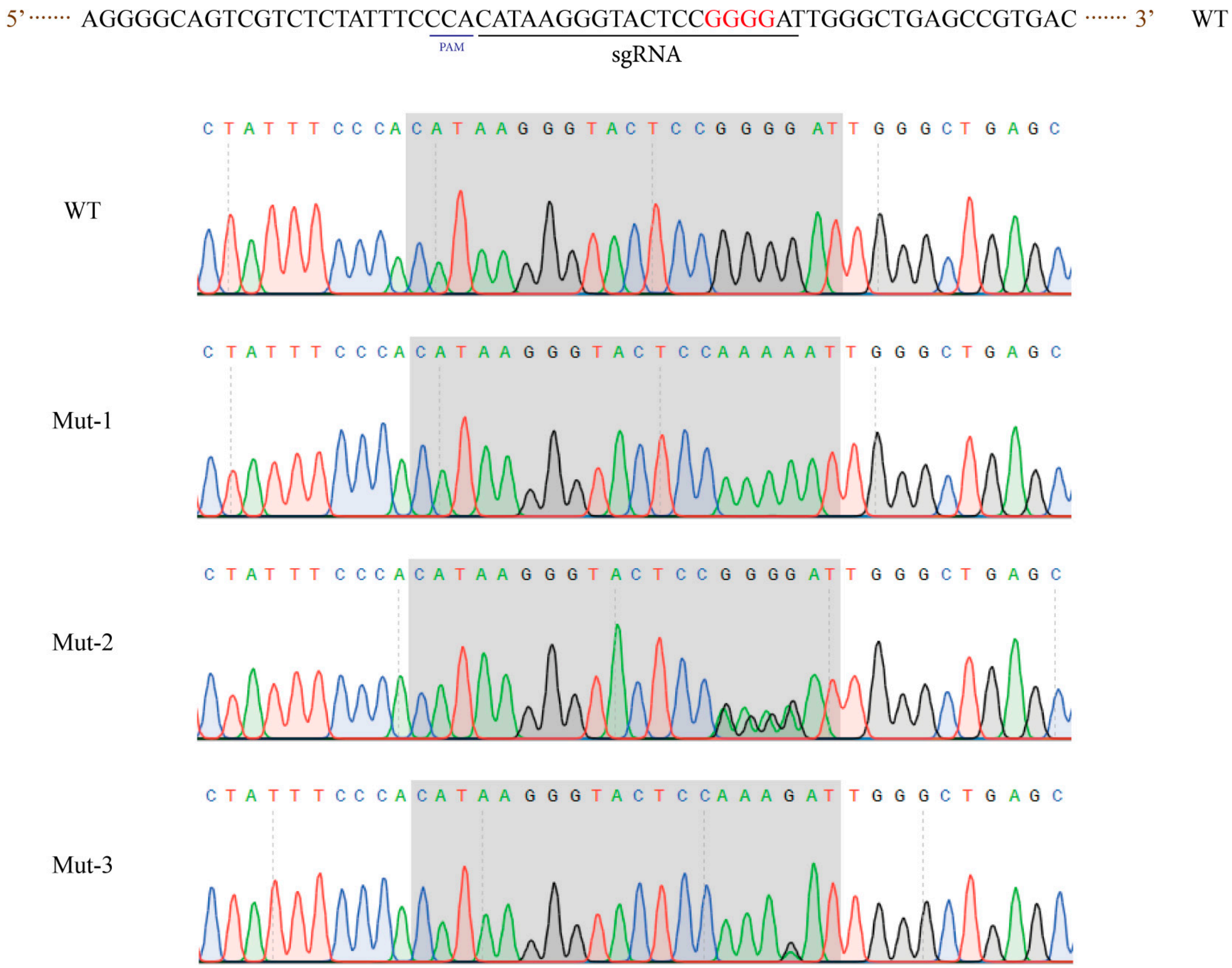

Figure S5. The sequence information of the Mut-1, Mut-2, and Mut-3. The shaded area refers to the sgRNA sequence.

a

#	Potential OTS	Sequence(5' to 3')	Indel
	CALR_A_521-sgRNA	ATCCCCGGAGTACCCTTATG TGG	
1	Potential OTS 1	GTCCTCGGAGGACCCTTGTG TGG	NO
2	Potential OTS 2	ATCCTCGGAGTGCGTTTATG GGG	NO
3	Potential OTS 3	AACACAGGACTACCCTTATG AGG	NO
4	Potential OTS 4	ATCTCCAGATTACCCTTCTG TGG	NO
5	Potential OTS 5	ATGCCAGGAGTACCTTTCTG TGG	NO
6	Potential OTS 6	ATCCTCGAAGTTCCCATATG GGG	NO

b

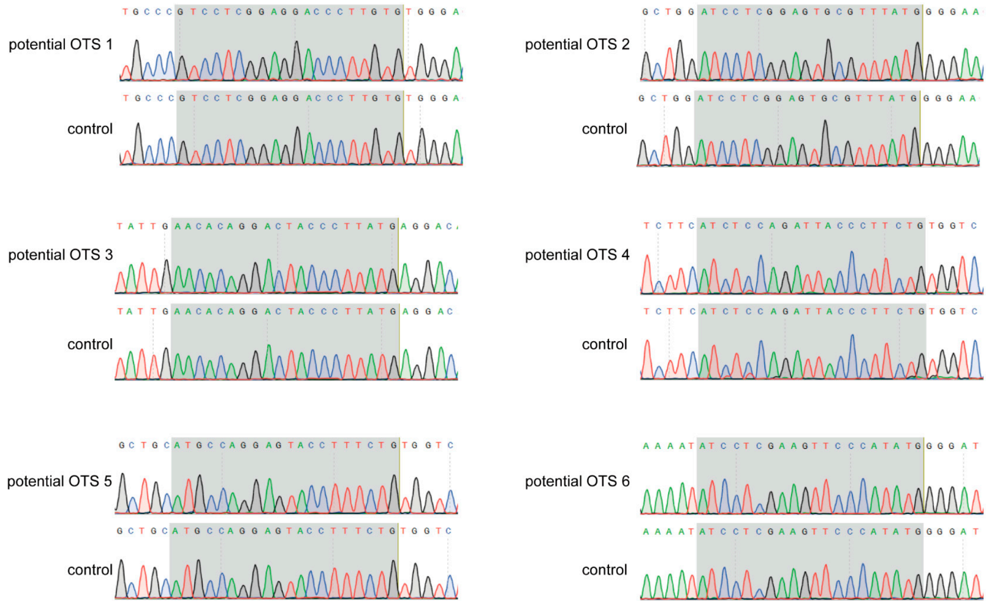

Figure S6. The evaluation of off-target effects of the sgRNA (CALR_A_521). **(a)** Six potential off-target sites were selected, and the off-target results were identified by PCR-sequencing. Blue letters indicate the PAM sequence. Red letters mark differences in sgRNA compared with the target sequence. The indel column shows the detected off-target results. **(b)** The sequence information for the potential off-target sites in Mut-1 and wild-type cells. The shaded area refers to the potential off-target site.
